# The Role of Lactate in Immune Regulation: A Metabolic Rheostat via Transporters, Receptors, and Epigenetic Modifiers

**DOI:** 10.3390/cells14141096

**Published:** 2025-07-17

**Authors:** Eun Jung Choi, Yoon Young Jang, Eun Joo Choi, Chang Joo Oh

**Affiliations:** 1 Department of Immunology, School of Medicine, Daegu Catholic University, Daegu 42472, Republic of Korea; 2 Department of Pediatrics, School of Medicine, Daegu Catholic University, Daegu 42472, Republic of Korea; 3 Department of Anesthesiology and Pain Medicine, W General Hospital, Daegu 42642, Republic of Korea

**Keywords:** lactate, anti-inflammation, immunometabolism

## Abstract

Lactate, once regarded as a metabolic byproduct, is now recognized as a critical immunometabolic regulator that shapes immune responses in both physiological and pathological contexts. This review examines how lactate accumulation occurs across diverse disease settings, including cancer, sepsis, and diabetes, through mechanisms such as hypoxia, mitochondrial dysfunction, and pharmacologic intervention. We then explore how lactate modulates immunity via four integrated mechanisms: transporter-mediated flux, receptor signaling (e.g., GPR81), context-dependent metabolic rewiring, and histone/protein lactylation. Particular emphasis is placed on the dichotomous effects of endogenous versus exogenous lactate, with the former supporting glycolytic effector functions and the latter reprogramming immune cells toward regulatory phenotypes via redox shifts and epigenetic remodeling. The review also highlights how the directionality of lactate transport, and the metabolic readiness of the cell determine, whether lactate sustains inflammation or promotes resolution. After analyzing emerging data across immune cell subsets and disease contexts, we propose that lactate serves as a dynamic rheostat that integrates environmental cues with intracellular metabolic and epigenetic programming. Understanding these context-dependent mechanisms is essential for the rational design of lactate-targeted immunotherapies that aim to modulate immune responses without disrupting systemic homeostasis.

## 1. Introduction

Lactate, the end-product of glycolysis, accumulates in rapidly proliferating or activated cells; typical representatives include cancer cells and immune cells [1]. In the immune system, lactate serves as a metabolic checkpoint that links intracellular energy pathways to immune cell fate and function.

Upon activation, both innate and adaptive immune cells undergo metabolic reprogramming. Pro-inflammatory M1 macrophages and activated effector T cells upregulate glycolysis and generate substantial amounts of lactate, whereas resting or regulatory cells such as M2 macrophages and regulatory T cells (Tregs) preferentially rely on oxidative phosphorylation [2,3]. These distinct metabolic profiles are not merely consequences of activation but play a crucial role in determining the magnitude and quality of immune responses.

Importantly, the immunological role of lactate is based on both intracellular production and extracellular accumulation. Lactate alters the direction of inflammatory responses, as well as redox and metabolic balance, within immune cells. Extracellularly, lactate released into the microenvironment, particularly by inflamed, tumoral, or ischemic tissues, can reach supraphysiological levels, acting as a paracrine signal that has a profound effect on immune cell behavior.

Lactate accumulates through diverse metabolic and environmental mechanisms across disease contexts (e.g., cancer, diabetes, and sepsis) [4,5,6]. In each of these settings, distinct factors, ranging from mitochondrial dysfunction and hypoxia to pharmacological inhibition or immune activation, increase lactate production in ways that are intimately linked to immunological outcomes. Therefore, understanding the origins of lactate accumulation is essential to interpreting its downstream effects on immune regulation.

In this review, we first examine how lactate accumulates across diverse pathological conditions, including cancer, diabetes, and sepsis, emphasizing how metabolic rewiring and environmental stress converge to elevate lactate levels. We then discuss how lactate modulates immune cell function through four interrelated mechanisms: membrane transport, receptor-mediated signaling, metabolism, and epigenetic. By integrating these disease-driven and cell-intrinsic perspectives, we aim to present a comprehensive framework for understanding lactate as a metabolic rheostat in immunity that is capable of both dampening and promoting immune responses depending on the cellular context and microenvironmental cues.

## 2. Results

### 2.1. Lactate Accumulation Across Diverse Pathophysiological Contexts

Lactate accumulates in various physiological and pathological states, each characterized by distinct underlying mechanisms [4,5,6,7]. While transient increases in lactate are a well-known physiological response to acute stress such as high-intensity exercise, this review focuses on conditions characterized by chronic or sustained lactate accumulation such as cancer, persistent inflammation, or metabolic disorders in which prolonged exposure to elevated lactate levels has a profound ability to reshape immune cell function and communication within the tissue microenvironment [7,8].

**Figure 1:**
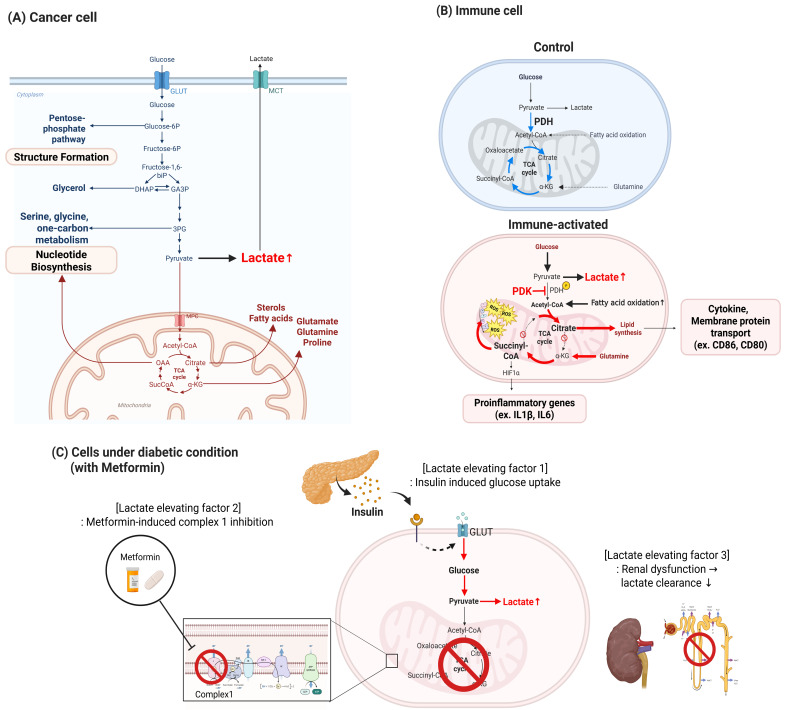
**Lactate-producing metabolic rewiring under cancer, immune, and diabetic conditions.** This figure illustrates how lactate accumulation arises through context-specific metabolic adaptations in (**A**) cancer cells, (**B**) immune cells, and (**C**) diabetic tissues exposed to pharmacologic or systemic stress. (**A**) Cancer cells rely on aerobic glycolysis (“Warburg effect”) wherein glucose is preferentially converted to lactate even in the presence of oxygen. This supports NAD^+^ regeneration and diverts glycolytic intermediates toward anabolic pathways including nucleotide biosynthesis (via serine/glycine and pentose phosphate pathways), amino acid synthesis (e.g., glutamine, proline), and lipid production. Mitochondrial export of citrate further fuels lipid biosynthesis, essential for rapid proliferation and structural expansion. (**B**) Immune cells, particularly macrophages upon activation, undergo a metabolic switch from oxidative phosphorylation to glycolysis. Pyruvate dehydrogenase kinase (PDK) inhibits pyruvate dehydrogenase (PDH), reducing mitochondrial pyruvate oxidation and leading to lactate accumulation. Citrate is used for lipid biosynthesis required for trafficking of membrane proteins (e.g., CD86, CD80), while succinate stabilizes HIF-1α, upregulating transcription of inflammatory genes (e.g., IL-1β, IL-6). This reprogramming supports immune effector function during inflammation. (**C**) Under diabetic conditions, lactate elevation results from three converging mechanisms: (1) Insulin-stimulated glucose uptake increases intracellular glucose and glycolytic flux, enhancing lactate production. (2) Metformin, a mitochondrial complex I inhibitor, impairs oxidative phosphorylation and shifts energy metabolism toward glycolysis. (3) Renal dysfunction reduces lactate clearance by limiting renal filtration and metabolic degradation. These combined effects lead to systemic lactate accumulation, independent of cellular proliferation or immune activation. Red arrows indicate metabolic flux toward lactate accumulation; mitochondrial substrates (e.g., acetyl-CoA, citrate, succinate) and signaling mediators (e.g., HIF-1α) are annotated to illustrate pathway alterations.

In the context of cancer, the tumor microenvironment (TME) represents a prototypical setting where chronic lactate accumulation profoundly influences immune regulation [9]. Within the TME, metabolic symbiosis—a cooperative metabolic exchange in which lactate produced by hypoxic cancer cells serves as a fuel for oxidative metabolism in neighboring stromal or cancer cells—emerges as a hallmark feature [10]. Proliferative cancer cells, characterized by high glycolytic flux and elevated lactate export, create an acidic extracellular milieu that directly shapes the metabolic fitness and effector functions of infiltrating immune cells [11]. Furthermore, stromal components—including cancer-associated fibroblasts and endothelial cells—contribute to lactate production and buffering capacity, reinforcing metabolic crosstalk that conditions immune responses to promote immune evasion [12]. These principles of lactate metabolism extend beyond cancer to other physiological and pathological contexts. Figure 1 illustrates three representative settings where lactate accumulation arises from distinct metabolic adaptations: proliferative cancer cells, activated immune cells, and diabetic tissues under pharmacological and systemic stress.

In cancer cells (Figure 1A), metabolic rewiring is characterized by aerobic glycolysis (i.e., the Warburg effect), in which glucose is preferentially converted to lactate even under normoxic conditions [6]. This facilitates sustained regeneration of NAD^+^ and permits diversion of glycolytic intermediates into anabolic pathways such as nucleotide, amino acid, and lipid biosynthesis, which are necessary for continuous cell proliferation and structural expansion [13,14].

In immune cells (Figure 1B), particularly activated macrophages, a similar shift to glycolysis occurs, facilitating rapid production of energy and supporting biosynthesis of cytokines and other immune effectors [1]. Pyruvate dehydrogenase kinase (PDK)-mediated inhibition of pyruvate dehydrogenase (PDH) blocks oxidation of mitochondrial pyruvate, resulting in lactate accumulation [15]. In mitochondria, this metabolic diversion truncates the TCA cycle, leading to the accumulation of distinct intermediates: citrate is exported to support membrane lipid biosynthesis for surface protein and cytokine trafficking, while succinate stabilizes HIF-1α and promotes transcription of inflammatory genes such as IL-1β [16,17].

A third and mechanistically distinct route to lactate elevation is observed under diabetic conditions (Figure 1C). First, insulin therapy promotes glucose uptake by increasing expression and translocation of GLUT4, thereby increasing glycolytic flux and favoring lactate production through elevated intracellular glucose metabolism [18]. Second, metformin, a widely used antidiabetic agent, inhibits mitochondrial complex I directly, thereby impairing oxidative phosphorylation (OXPHOS) and reducing ATP production via the electron transport chain [19]. As a result, cells increasingly rely on anaerobic glycolysis to meet their energy demands, leading to increased conversion of pyruvate to lactate by lactate dehydrogenase (LDH). Third, renal dysfunction in diabetes compromises lactate clearance by impairing both its filtration and metabolic degradation in the kidney, thereby contributing to systemic accumulation of lactate [20].

**Figure 2:**
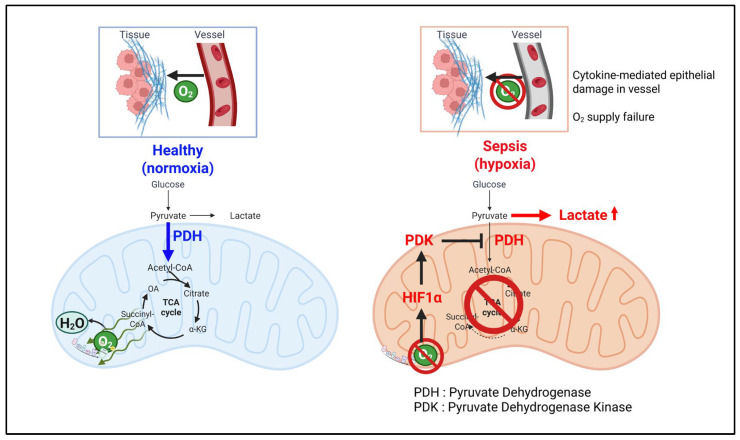
**Hypoxia-driven accumulation of lactate during sepsis** in healthy tissue under normoxic conditions, glucose-derived pyruvate is oxidatively metabolized via the mitochondrial TCA cycle following conversion to acetyl-CoA by the pyruvate dehydrogenase (PDH) complex. This supports efficient production of ATP via oxidative phosphorylation. During sepsis, cytokine-induced vascular injury leads to endothelial damage and impaired oxygen delivery to surrounding tissues. The resulting hypoxic microenvironment stabilizes hypoxia-inducible factor 1α (HIF-1α), which upregulates pyruvate dehydrogenase kinase (PDK). PDK phosphorylates and inhibits PDH, preventing pyruvate from entering the TCA cycle. Instead, pyruvate is converted preferentially to lactate via lactate dehydrogenase (LDH), resulting in a marked increase in systemic lactate levels. This metabolic shift is distinct from the lactate-producing rewiring observed in proliferating tumor or immune cells and reflects a stress-driven response to oxygen deprivation during acute inflammation.

While lactate production at the cellular level often reflects immune cell–specific metabolic rewiring to support proliferation or effector functions, systemic lactate accumulation, particularly in acute inflammatory conditions such as sepsis, is primarily driven by tissue hypoxia. In sepsis, pro-inflammatory cytokines induce widespread endothelial damage, impairing oxygen delivery across multiple organs. This results in the stabilization of hypoxia-inducible factor 1α (HIF-1α), which upregulates PDK [21,22,23]. PDK inhibits PDH, thereby suppressing oxidation of mitochondrial pyruvate and diverting pyruvate toward lactate production via lactate dehydrogenase (LDH) [7,24]. Consequently, not only immune cells, but virtually all parenchymal cells, shift toward anaerobic glycolysis, collectively amplifying systemic lactate levels (Figure 2).

### 2.2. Lactate Influences Immunity Through Distinct but Integrated Mechanistic Layer

**Figure 3:**
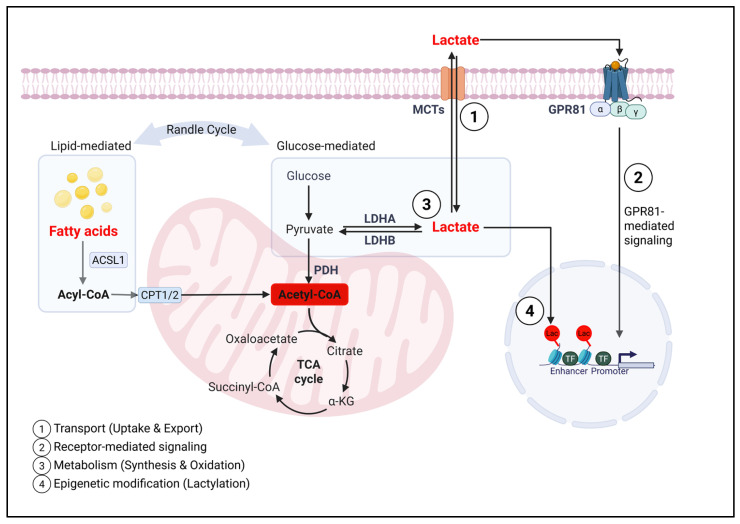
**Multilevel mechanisms of lactate-mediated immune modulation.** Lactate exerts pleiotropic effects on immune cell function through four interconnected mechanisms. (1) Transport: Lactate is imported and exported by monocarboxylate transporters (MCTs), particularly MCT1 and MCT4, thereby regulating intracellular levels in a context-dependent manner. (2) Receptor-mediated signaling: Extracellular lactate activates the G protein–coupled receptor GPR81 (HCAR1), which signals through Gi to suppress cAMP and downstream pro-inflammatory pathways. (3) Metabolic modulation: Lactate competes with glucose- and fatty acid-derived metabolites for entry into mitochondrial metabolisms, influencing PDH activity and acetyl-CoA levels. (4) Epigenetic regulation: Intracellular lactate can donate its acyl group for histone lactylation, thereby modifying enhancer/promoter accessibility and gene expression patterns, particularly in inflammatory or suppressive immune environments. These converging mechanisms position lactate as a central immunometabolic rheostat that integrates extracellular conditions with intracellular immune programming.

Having outlined the diverse mechanisms by which lactate accumulates at both cellular and systemic levels across different pathophysiological contexts, we now turn to the mechanism by which increased lactate levels modulate immune cell behavior. Lactate is increasingly recognized not merely as a metabolic byproduct but as a bona fide immunometabolite and signaling molecule. Rather than passively reflecting metabolic stress or adaptation, it actively shapes immune responses through multiple layers of regulation, including membrane transport, receptor-mediated signaling, intracellular metabolic shifts, and epigenetic modification. In the following section, we examine these mechanistic pathways in detail, highlighting how lactate serves as a metabolic rheostat that fine-tunes immune cell function and fate (Figure 3).

To provide an integrated overview, we categorize the immunoregulatory roles of lactate into four mechanistic tiers. First, lactate is exchanged across immune cell membranes via monocarboxylate transporters (MCTs), with the direction of transport varying according to activation status and microenvironmental lactate gradients. Second, extracellular lactate functions as a signaling molecule through G protein–coupled receptors (GPCRs) such as GPR81, thereby altering intracellular second messengers and inflammatory gene expression. Third, lactate intersects with core intracellular metabolic pathways, competing with other substrates, shaping redox balance, and influencing mitochondrial function. Fourth, lactate acts as an epigenetic modifier via histone and protein lactylation, linking metabolic status to gene regulation in immune cells. These layers operate in concert to integrate environmental signals with cellular programming and are discussed in the sections that follow.

#### 2.2.1. Transporters and Lactate Flux: Role of MCTs in Immunity

Movement of lactate across cell membranes is mediated by MCTs, primarily MCT1, which is encoded by SLC16 genes [25,26]. Immune cells modulate the expression of MCTs in tune with their metabolic programs, thereby controlling lactate export and import [1]. Changes in transporter expression during immune activation or differentiation alters cell function by influencing cellular lactate accumulation, pH, and the availability of lactate as a fuel or signaling molecule (Table 1).

- MCT1

MCT1 (SLC16A1) is a high-affinity monocarboxylate transporter that facilitates bidirectional lactate transport depending on concentration and proton gradients [34]. It is expressed by various immune cells and plays a critical role in modulating immune responses by regulating availability of lactate for metabolism and by maintaining intracellular pH. In lactate-rich or glucose-depleted environments, MCT1 supports lactate uptake for oxidative metabolism, whereas in highly glycolytic cells, it contributes to lactate efflux to prevent acidification and sustain glycolytic flux [35].

Effector T cells: MCT1 is upregulated during activation of CD8^+^ T cells to export lactic acid produced during high-rate glycolysis, maintaining intracellular pH and enabling sustained glycolytic activity [27]. In lactate-rich environments, insufficient export leads to a drop in pH that (1) inhibits key glycolytic enzymes directly, thereby reducing flux, and (2) causes feedback inhibition on glycolysis, which disrupts nucleotide synthesis and halts proliferation, ultimately pushing cells toward functional exhaustion rather than memory formation [27,35].

Effector B cells: MCT1 is upregulated during B cell activation to accommodate increased glycolytic flux and facilitate lactate export [31]. Deficiency of MCT1 impairs lactate efflux, disrupts glycolysis and nucleotide biosynthesis, and leads to reduced B cell proliferation and antibody production, highlighting its critical role in metabolic support of humoral immunity [31].

Regulatory T cells (Tregs): Tumor-infiltrating Tregs express high levels of MCT1, which enables lactate uptake and conversion via lactate dehydrogenase-B (LDHB) to support oxidative metabolism. This allows Tregs to maintain proliferation and suppressive functions in glucose-depleted, acidic environments. Depleting MCT1 selectively impairs the accumulation of Tregs in tumors, thereby enhancing anti-tumor immunity [29].

Dendritic cells (DCs): DCs express MCT1, which facilitates uptake of extracellular lactate, particularly under inflammatory conditions [33]. In a cervical cancer model, silencing of MCT1 in DCs mitigates the immunosuppressive effects of lactate exposure, preserving surface activation markers and pro-inflammatory cytokine secretion upon LPS stimulation [33].

Natural Killer cells (NKs): NK cells express MCT1 (and possibly MCT4). Blocking MCT1 alleviates tumor-derived lactate-induced dysfunction by limiting lactate influx, thereby improving cytotoxicity [36].

- MCT4

MCT4 (SLC16A3) is a low-affinity, high-capacity lactate transporter induced by HIF-1α in highly glycolytic cells [37]. Unlike MCT1, which facilitates bidirectional lactate transport depending on concentration and proton gradients, MCT4 functions predominantly as an efflux transporter, optimized for sustained lactate export in cells with high glycolytic flux. This helps to prevent intracellular acidification and allows glycolysis to proceed at a high rate, i.e., its maintains the pro-inflammatory status of M1 cells [37]. In pro-inflammatory macrophages, MCT4 is upregulated alongside lactate dehydrogenase-A (LDHA) [32,38]. Genetic deletion of MCT4 from inflammatory macrophages increases intracellular lactate levels, enhances H3K18 lactylation, and promotes expression of reparative M2 and TCA cycle genes, thereby shifting the macrophage phenotype toward inflammation resolution and reducing atherosclerosis (i.e., M2) [32]. Naïve T cells express very low levels of MCT1 and MCT4, but activated T cells show a marked and sustained upregulation of MCT4, supporting lactate efflux to help maintain glycolytic flux [28].

- MCT11

Certain MCTs are upregulated selectively in specific immune cell populations, in which they play distinct functional roles aligned with cellular metabolic status. Recent findings by Frisch et al. demonstrate that MCT11 (SLC16A11) is upregulated selectively in terminally exhausted CD8^+^ T cells (Tex) within tumors and at sites of chronic infection, where it facilitates uptake of lactic acid [30]. This uptake increases the intracellular acid load and disturbs the redox balance, thereby reinforcing Tex dysfunction by promoting mitochondrial depolarization, reducing ATP production, and increasing expression of inhibitory receptors [30].

Although extracellular lactate levels are elevated in metabolically active immune environments, expression of MCTs is governed primarily by upstream regulatory factors such as HIF-1α, inflammatory mediators, and activation-induced transcriptional programs rather than by lactate itself [1,3]. HIF-1α induces robust expression of MCT4 (SLC16A3) in myeloid cells during pro-inflammatory activation, thereby promoting lactate export to sustain glycolysis [32]. Conversely, anti-inflammatory cytokines such as IL-10 may attenuate expression of MCT4 as cells transition toward oxidative metabolism, although direct evidence remains limited [39]. MCT1 (SLC16A1), while often constitutively expressed in T cells, is further upregulated by activation pathways such as c-Myc and CD28 signaling to accommodate increased metabolic flux [27].

#### 2.2.2. Lactate-Responsive Receptors in Immune Modulation

In addition to its intracellular metabolic effects, lactate also acts as an extracellular signaling molecule that shapes immune cell behavior through receptor-mediated pathways. Circulating lactate can bind to specific GPCRs on cell surfaces, thereby initiating signaling cascades that alter immune cell behavior [40]. The most extensively characterized lactate receptor is GPR81 (HCAR1), a Gi/o-coupled GPCR for L-lactate that is expressed in adipose tissue, skeletal muscle, and leukocyte populations, where its activation typically suppresses intracellular cAMP levels [41]. In adipose tissue, GPR81 suppresses lipolysis via a Gi-coupled mechanism that reduces intracellular cAMP levels and inhibits protein kinase A-mediated phosphorylation of key lipolytic enzymes [42].

- GPR81

In immune cells, GPR81 is increasingly recognized as a metabolic sensor that links extracellular lactate accumulation to immunoregulatory outcomes. Lactate promotes macrophage polarization toward an M2 phenotype via GPR81 signaling under conditions of LPS stimulation, a shift characterized by increased expression of Arg1, CD206, and IL-10, and reduced expression of iNOS and TNF-α, thereby contributing to tissue repair and immunosuppressive outcomes in sepsis [43,44]. Activation of GPR81 recruits β-arrestin 2 (ARRB2), which suppresses phosphorylation of NF-κB and downregulates transcription of Il1b, Nlrp3, and Casp1, thereby attenuating maturation and secretion of IL-1β in models of TLR4-mediated inflammation [45]. More recent studies support these findings. For example, lactate inhibits LPS-induced expression of IL-12 and IL-6 in macrophages via GPR81-dependent activation of a signaling cascade involving AMP-activated protein kinase (AMPK) and downstream effectors that limit NF-κB translocation [46]. In the tumor microenvironment, elevated lactate levels likely engage GPR81 on DCs to induce a state of “immune paralysis”, characterized by reduced production of IL-12 and impaired T cell priming capacity [47,48]. Recent findings have revealed that GPR81 expressed on dendritic cells within the tumor microenvironment suppresses MHC II surface expression and cytokine production, thereby impairing antigen presentation and dampening T cell activation [49]. These data underscore a paracrine immunosuppressive function of lactate-GPR81 signaling beyond the tumor cell-autonomous effects previously described. Beyond its role in macrophages and dendritic cells, lactate critically modulates myeloid-derived suppressor cells (MDSCs) [50]. For instance, in pancreatic cancer, lactate enhances MDSC immunosuppressive activity via the GPR81–mTOR–HIF-1α–STAT3 axis, promoting tumor immune evasion and radioresistance [51].

- Proton-sensing receptors (GPR65/GPR4/GPR132)

Although GPR65, GPR4, and GPR132 sense acidic pH rather than lactate per se, they are highly relevant in lactate-rich, acidic microenvironments [52]. Although these receptors do not bind lactate directly, they complement GPR81 by responding to the downstream consequences of lactate accumulation (i.e., acidosis). In an acidic-inflamed tissue, lactate can simultaneously activate GPR81 (specific lactate signal) and GPR65/132 (general acid stress signals) to orchestrate a complex immune response.

GPR65 (TDAG8) is a proton-sensing GPCR that is activated under conditions of extracellular acidosis, in where it signals through Gs protein to elevate intracellular cAMP, broadly contributing to immunosuppression in acidic, lactate-rich microenvironments [53]. In macrophages, activated GPR65 suppresses pro-production of pro-inflammatory cytokines such as TNF-α and IL-6 via cAMP signaling [54]. In T cells, GPR65 deficiency increases the expression of Th2 cytokines, and exacerbates skin inflammation in vivo, confirming its T cell-intrinsic immunosuppressive function [55].

GPR132, a proton-sensing GPCR expressed by macrophages, mediates chemotaxis toward acidic, lactate-rich microenvironments enriched in dying cells by sensing lactate-induced acidification; this activates the MyD88–PI3K–AKT–MMP9 signaling axis and not only facilitates clearance of necrotic debris but also reprograms macrophages toward an M2-like tumor-promoting phenotype, characterized by increased motility, phagocytosis, and expression of metabolic genes (e.g., Hif1α, Ldha) and immunoregulatory markers [52,56].

In parallel, lactate also functions via autocrine and paracrine mechanisms mediated by GPR81 and extracellular acidification in the tumor microenvironment [57]. The acidic pH resulting from lactic acid accumulation creates an inward proton gradient that drives proton-coupled nutrient transporters (such as PEPT1/PEPT2 and folate symporters), potentially affecting peptide uptake during infection or folate-dependent proliferation in activated macrophages and T cells [57].

- NDRG3

In addition to the membrane-bound receptor, recent studies have identified NDRG3 (N-myc downstream-regulated gene 3) as a cytosolic lactate sensor that activates the cRaf–ERK pathway upon lactate binding, thereby promoting cell survival and angiogenic gene expression under hypoxia [58]. In immune cells, particularly tumor-associated macrophages, this NDRG3–Raf–ERK axis complements cytokine signaling and contributes to metabolic polarization by enhancing Arg1 and Vegfa expression in lactate-rich, hypoxic tumor regions [59,60]. These findings suggest that intracellular lactate sensing through NDRG3 plays a non-redundant role alongside GPR81-mediated signaling in modulating immune cell phenotypes in metabolically stressed microenvironments.

#### 2.2.3. Lactate Synthesis and Oxidation, and Metabolic Consequences in the Immune System

A hallmark of immune cell activation is a metabolic shift toward glycolysis, resulting in lactate accumulation [61]. Classically activated macrophages (i.e., M1) provide a clear example: upon stimulation by LPS or IFN-γ, macrophages increase glucose uptake and glycolysis markedly, with pyruvate being preferentially converted to lactate by LDHA [62]. This helps regenerate NAD^+^ for continued high-rate glycolysis, but leads to an acidic, lactate-rich microenvironment. By contrast, alternatively activated “M2” macrophages (involved in resolution and tissue repair) rely more on oxidative phosphorylation and fatty acid oxidation, which generate less lactate. Indeed, as macrophages transition from an M1 to an M2 state, their mitochondrial metabolism is restored and lactate production declines [63].

Having previously reviewed the roles of lactate uptake via MCTs and lactate-induced signaling through GPR81 and other GPCRs, this section will now focus on how lactate metabolism itself, via modulation of enzymatic expression and activity, shapes immune cell phenotype and function. We discuss key regulators such as LDH and PDH and examine how their dynamic regulation determines whether lactate acts as a byproduct of glycolysis or as a mitochondrial fuel, ultimately dictating the inflammatory or suppressive outcome of immune responses.

- LDH

The distinct roles of LDH isoforms in immune cells are an area of active investigation. LDHA predominantly catalyzes pyruvate → lactate, while LDHB catalyzes the reverse process (lactate → pyruvate) to feed the TCA cycle [62]. Typically, pro-inflammatory macrophages upregulate LDHA, thereby reinforcing lactate production [64,65]. Enforcement of a fragmented mitochondrial phenotype in macrophages increases LDHA activity and lactate production, which promotes histone lactylation and upregulation of M2 markers such as arginase-1 and arginase-2 [63,66]. In parallel, the downregulation of LDHB in macrophages increases lactate accumulation and promotes histone lactylation, thereby facilitating M2-like polarization and tumor-associated macrophage formation within the tumor microenvironment [67].

T cells also undergo metabolic remodeling, which affects lactate production. A recent study demonstrated that effector T cells acquire an LDHA-dominant profile, with the LDHA/LDHB ratio increasing by up to 3.3-fold, a process driven by elevated Myc signaling and epigenetic remodeling (e.g., histone acetylation) at the LDHA locus; this reprogramming increases aerobic glycolysis to enable rapid production of ATP as well as generation of biosynthetic intermediates required for proliferation and effector function [68]. Similarly, enforced expression of LDH isoforms can affect T cell fate: a recent report indicates that overexpression of LDHB (which enhances lactate-to-pyruvate flux) in tumor-specific CD8^+^ T cells improves their function in the lactate-rich tumor microenvironment [68,69].

- PDH

PDH is a gatekeeper enzyme complex that commits pyruvate to the TCA cycle by converting it to acetyl-CoA [70]. In activated immune cells, PDH activity is suppressed by PDK-mediated phosphorylation, diverting pyruvate from mitochondrial oxidation toward lactate production [39,71]. This is evident in inflammatory macrophages in which PDH is inhibited, thereby contributing to the broken TCA cycle and citrate accumulation (supporting fatty acid synthesis and itaconate production), both of which are characteristic of M1 polarization [39,71,72]. When inflammation resolves, PDH is reactivated (and PDK is downregulated), allowing lactate and pyruvate to be oxidized and enabling the cell to transition to an OXPHOS-dependent state [72,73].

In T cells, inhibition of PDH by PDKs promotes effector differentiation, while sustained PDH activity favors development of memory T cells and Tregs [71,74,75]. In murine models, deletion or pharmacologic inhibition of PDK1 diverts pyruvate into the TCA cycle at the expense of lactate production, thereby skewing T cell differentiation away from Th17 and towards Tregs [74].

In summary, immune activation is tightly linked to altered lactate production. Enzymatic regulators (i.e., LDH and PDH) control whether pyruvate is diverted to lactate or oxidized, thereby determining cell phenotype. Pro-inflammatory states favor a high-lactate, high-LDHA metabolism state, which can feed forward into signaling and epigenetic changes that eventually dampen inflammation or support immunosuppressive phenotypes. By contrast, immune quiescence or certain regulatory states involve ramping down glycolysis and lactate output. The balance between these metabolic pathways is a crucial aspect of immune regulation, making them attractive targets for intervention (e.g., using LDH inhibitors or metabolic modulators to skew immune responses).

- Impact of lactate-driven NAD^+^/NADH balance on immune cell function

**Figure 4:**
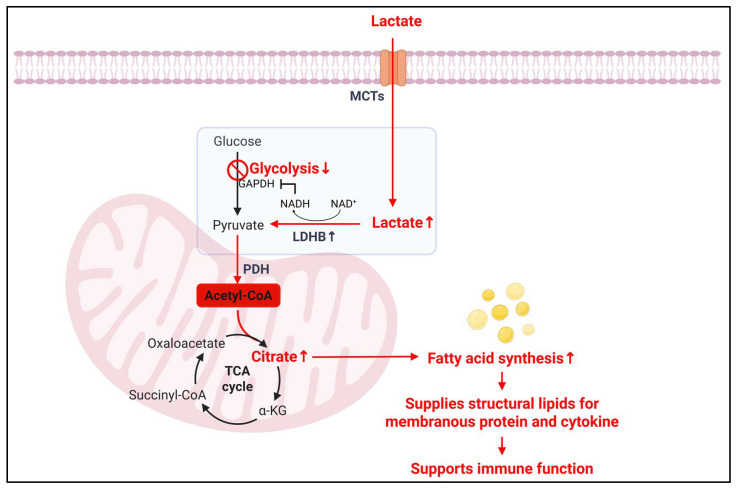
**Lactate-induced modulation of lipid metabolism in immune cells.** Extracellular lactate is imported into immune cells via MCTs and converted to pyruvate by LDHB, a process that consumes NAD^+^ and generates NADH. The resulting redox imbalance inhibits GAPDH activity, thereby suppressing glycolysis. Pyruvate enters the mitochondria and is converted to acetyl-CoA by PDH, fueling the TCA cycle and increasing citrate levels. Citrate is exported to the cytosol and converted to acetyl-CoA for fatty acid synthesis, which supplies structural lipids required for membrane formation and cytokine secretion. This lipid anabolic process supports immune cell function, particularly in environments where glucose is limited and lactate is abundant.

Lactate metabolism in immune cells modulates redox state and metabolic integration, directly impacting their capacity for proliferation, lineage commitment, and effector responses. Interconversion of pyruvate and lactate via LDH isoforms is tightly coupled to NAD^+^/NADH redox pair, with LDH-A-mediated lactate production oxidizing NADH to NAD^+^ and LDH-B-mediated lactate consumption reducing NAD^+^ to NADH [62]. The cytosolic NAD^+^/NADH ratio critically regulates glycolytic throughput, particularly through NAD^+^-dependent enzymes such as glyceraldehyde-3-phosphate dehydrogenase (GAPDH), which catalyzes a rate-limiting oxidation step (Figure 4) [28,76,77]. For example, activated T cells rely on continuous regeneration of NAD^+^ to sustain glycolysis and serine synthesis; however, depletion of NAD^+^ from lactate-rich environments disrupts these pathways, thereby impairing nucleotide production and T cell proliferation [76].

- Impact of lactate on amino acid metabolism in immune cells

Lactate accumulation influences amino acid metabolism, reshaping immune function within inflamed or tumor microenvironments [78]. Elevated lactate stabilizes hypoxia-inducible factor-1α (HIF-1α), which transcriptionally upregulates arginase-1 (Arg1) while suppressing inducible nitric oxide synthase (iNOS), thereby diverting arginine metabolism away from nitric oxide (NO) production toward ornithine and polyamine biosynthesis [79]. This metabolic shift not only dampens pro-inflammatory M1 macrophage responses but also increases intracellular ornithine availability, which facilitates downstream pathways essential for cellular proliferation and tissue repair, reinforcing a tissue-repairing M2-like phenotype [80].

In lactate-rich and glucose-poor environments, such as the tumor microenvironment, immune cells increasingly rely on glutamine-derived α-ketoglutarate to support tricarboxylic acid (TCA) cycle anaplerosis, sustaining mitochondrial function despite limited glycolytic input [81]. This metabolic adaptation is essential for maintaining both mitochondrial integrity and NADPH regeneration in lactate-exposed macrophages [81]. However, when glutamine becomes limited—due to tumor consumption or competitive uptake—T cells and M1 macrophages experience bioenergetic failure, reduced proliferation, and impaired cytokine production [78]. Glutamine also serves as a key substrate for hexosamine biosynthesis, yielding UDP-N-acetylglucosamine (UDP-GlcNAc); in lactate-rich environments, M2 macrophages increasingly depend on glutamine to sustain the glycosylation of immunoregulatory receptors such as CD206 and PD-L2, thereby maintaining their suppressive phenotype [82].

- Impact of lactate on lipid synthesis and consequences in immune cells

Lactate metabolism in immune cells intersects with lipid metabolism at multiple levels. In lactate-rich environments, CD4^+^ T cells shift toward fatty acid synthesis (FAS), a process supported by SREBP1 and mTORC1 signaling, which facilitates membrane biogenesis and Th1 effector expansion [4,7]. Lactate-derived pyruvate fuels the TCA cycle and contributes to the accumulation of cytosolic citrate, which is cleaved by ATP-citrate lyase to generate acetyl-CoA for de novo lipogenesis and cholesterol synthesis (Figure 4). This mechanism supports the metabolic needs of proliferating immune cells, including dendritic cells (DCs) and macrophages, during inflammation [83].

In melanoma models, tumor-derived lactate activates SREBP2 in DCs, upregulating cholesterol biosynthesis genes and driving a regulatory, tolerogenic phenotype (“mregDCs”). Inhibiting lactate uptake in this context prevents lipid overload and enhances the immunostimulatory capacity of DCs [84].

- Impact of lactate on reactive oxygen species (ROS) signaling and redox balance in immune cells

The role of lactate in redox biology is complex and highly context-dependent, exhibiting both antioxidant and pro-oxidant properties depending on the metabolic and signaling environment. Lactate is considered to have antioxidant properties, as it may promote NADH oxidation via the LDH reaction, thereby regenerating NAD^+^ and potentially reducing superoxide generation from an over-reduced electron transport chain [85]. However, emerging evidence indicates that lactate may also promote oxidative stress under certain metabolic conditions. Exposure to lactate induces mitochondrial fission through ERK and DRP1 signaling, leading to increased mitochondrial ROS production and activation of pro-fibrotic gene expression via NF-κB and stabilization of HIF-1α [86].

In immune cells, lactate-induced ROS can also have dual effects. On the one hand, moderate ROS levels are essential for signaling and functional activation [87]. On the other, excessive ROS may activate the inflammasome, apoptosis, or senescence [88,89]. In inflamed, lactate-rich tissues, macrophages may shift toward an M2-like phenotype, a process driven by ROS and HIF-1α-dependent upregulation of VEGF and Arg1 [90]. Further compounding its redox-modulatory role, lactate increases mitochondrial ROS production by promoting oxidative dimerization of prolyl hydroxylase domain protein 2 (PHD2), thereby amplifying HIF-1α activity in a feedforward loop [91,92,93]. Collectively, these findings highlight lactate as a metabolite that shapes immune outcomes through redox-sensitive signaling and mitochondrial regulation.

#### 2.2.4. Epigenetic Effects of Lactate: Histone and Protein Lactylation

A pivotal discovery in the field of immunometabolism revealed that lactate, a metabolic byproduct, can induce lysine lactylation (Kla), a novel post-translational modification, of histones and other proteins, thereby altering the epigenetic and signaling landscape of immune cells directly [63]. Histone and non-histone protein lactylation offer a mechanistic link between glycolytic metabolism and gene regulation, enabling lactate to shape immune cell function by influencing macrophage polarization, T cell differentiation, and the balance between immune activation and suppression [94,95,96].

- Histone lactylation

A prototypical example of the epigenetic influence of lactate is macrophage polarization. Zhang et al. showed that during prolonged stimulation with LPS, lactate accumulated in M1 macrophages induces histone lactylation, particularly H3K18la at the Arg1 promoter, which activates expression of M2-like genes as a feedback mechanism to promote resolution of inflammation [63]. Thus, lactate produced by highly glycolytic M1 macrophages serves as a feedback signal to trigger an epigenetic switch, turning on a reparative program to resolve inflammation.

Exogenous lactate increases CD8^+^ T cell “stemness” by fueling mitochondrial oxidative metabolism and increasing intracellular pools of acetyl-CoA and lactyl-CoA, which in turn drive histone lactylation at the promoters of TCF-1-regulated genes involved in self-renewal and memory potential, thereby epigenetically reinforcing a stem-like transcriptional program [97]. Upon activation, CD8^+^ T cells rapidly increase histone lactylation at H3K18 and H3K9, which then enhances chromatin accessibility at loci encoding immunoregulatory and tissue-repair genes such as *Il10* and *Areg*, thereby epigenetically promoting a reparative transcriptional program during the effector-to-memory transition [98]. Increasing histone lactylation (e.g., by adding lactate or inhibiting histone deacetylases [HDACs] that might remove lactyl groups) tends to boost expression of genes associated with effector function (i.e., *IFNG* encoding IFN-γ or *PRDM1* encoding Blimp-1 in certain contexts); however, it also boosts expression of genes associated with persistence (i.e., *TCF7* for TCF-1 in stem-like cells) [98].

- Non-histone protein lactylation

Lactylation is not limited to histones; numerous cytoplasmic and nuclear proteins are also modified under high-lactate conditions [99,100]. For example, glycolytic enzymes such as GAPDH and aldolase undergo lactylation, which may reduce their activity and act as a feedback brake on glycolysis [101,102].

A study in a Parkinson’s disease model showed that glycolytic microglia produce lactate, which lactylates NF-κB p65 and increases transcription of pro-inflammatory genes, thereby contributing to neurodegeneration [103]. This finding underscores the notion that lactylation amplifies inflammation in a disease-specific context. Similarly, in LPS-stimulated macrophages, lactate induces p300/CBP-mediated lactylation of HMGB1 at lysine-30, which interferes with nuclear retention by competing with acetylation [104]. During sepsis, this modification facilitates exosomal release of HMGB, which promotes vascular inflammation and endothelial barrier disruption [104]. Additionally, hypoxia-induced accumulation of lactate in murine muscle cells promotes lactylation of the PDH E1 subunit, leading to activation of PDH and stabilization of HIF-1α through reduced α-ketoglutarate-dependent PHD activity, a mechanism that may also operate in immune cells under similar metabolic stress [105].

## 3. Discussion

The immunoregulatory roles of lactate cannot be interpreted through a single mechanism or universal model. This is because its effects are highly context-dependent, shaped not only by concentration gradients and transporter expression, but also by metabolic status and the differentiation programs particular to each immune cell. While MCT1 is often described as a lactate importer, its directionality is governed not by the transporter itself but by transmembrane proton–lactate gradients and intracellular metabolism. In glycolytic cells such as activated effector T cells, MCT1 may function predominantly as an exporter [27,28]. By contrast, oxidative cells such as Tregs or M2 macrophages utilize MCT1 for lactate uptake to support mitochondrial metabolism and immunosuppressive function [29]. Therefore, MCT expression alone provides limited predictive value. Without considering metabolic flux and directionality, reliance on transporter expression alone may lead to misleading immunological interpretations or therapeutic expectations.

This complexity becomes particularly relevant in the context of MCT1 inhibition. The same inhibitor can yield opposing immunologic effects depending on cell type. In Tregs, for example, blocking MCT1 deprives cells of lactate as fuel, thereby diminishing their suppressive capacity and strengthening anti-tumor immunity [29]. By contrast, effector T cells, which rely on glycolysis, may be less affected (or even impaired) because of intracellular acidification [27]. DCs also show functional recovery upon removal of tumor-derived lactate via MCT1 blockade, suggesting that lactate suppresses not only the function of T cells but also that of antigen-presenting cells [33,106]. These findings imply that inhibiting MCT1 though nonspecific methods may cause a selective shift in the tumor immune landscape by disrupting access of lactate to immunosuppressive cell types.

The dual identity of lactate, i.e., as both a metabolic fuel and signaling molecule, adds further complexity. Through activation of GPR81, lactate suppresses intracellular cAMP and pro-inflammatory transcriptional programs [40,41,42]; however, this receptor-mediated signaling also inhibits lipolysis, which may not align with the metabolic needs of M2 macrophages that require fatty acid oxidation. These observations suggest that GPR81-driven anti-inflammatory effects might, at least in part, be mediated through transcriptional mechanisms rather than reflecting direct support for underlying metabolic demands [43,44,45,46]. Meanwhile, MCT1-mediated uptake of lactate promotes mitochondrial metabolism and histone lactylation, offering an alternative route to anti-inflammatory programming that is mechanistically distinct from GPR81 signaling [29,32,98].

In parallel, lactate may modulate immune function by competing with fatty acid oxidation. Although direct evidence in immune cells is limited, lactate-driven mitochondrial entry, as well as GPR81-mediated suppression of lipolysis, support the concept that lactate and fatty acids compete for metabolic dominance, a relationship illustrated in Figure 3. This concept is reminiscent of the classical Randle cycle, in which glucose and fatty acid oxidation inhibit each other by competing for substrates at the mitochondrial level [107,108]. In lactate-rich environments, such competition may favor regulatory or reparative immune phenotypes while at the same time limiting effector functions.

Importantly, the immunologic effects of lactate are shaped not only by cellular context but also by its source, i.e., produced intracellularly or imported from the extracellular environment. In activated immune cells, particularly effector CD8^+^ T cells, lactate is generated endogenously via LDHA during aerobic glycolysis. This process facilitates regeneration of NAD^+^ from NADH, thereby sustaining GAPDH activity, glycolytic flux, and pro-inflammatory functions such as cytokine secretion and proliferation [28,62,76]. Under these conditions, lactate functions as a metabolic enabler that supports short-term effector responses.

By contrast, in inflamed or tumor microenvironments where extracellular lactate is abundant, uptake via MCT1 leads to conversion of lactate to pyruvate by LDHB, a reaction that generates NADH and lowers the intracellular NAD^+^/NADH ratio. This redox imbalance can impair glycolysis at NAD^+^-dependent steps such as GAPDH, thereby limiting energy production and biosynthetic capacity [76,98]. In this context, exogenous lactate may suppress effector functions, particularly in cells already operating at high glycolytic capacity.

This redox-based dichotomy also affects epigenetic regulation. Recent studies show that exposure of glycolytic effector CD8^+^ T cells to exogenous lactate does not further increase histone lactylation, indicating that a saturation point is likely reached via intrinsic lactate production [98]. By contrast, naïve and memory CD8^+^ T cells, which are less glycolytic and rely more on oxidative metabolism, readily import extracellular lactate via MCT1. In these cells, lactate increases histone lactylation at residues such as H3K18 and H3K9, thereby promoting transcriptional programs associated with self-renewal, persistence, and long-term immune surveillance [98].

These contrasting responses illustrate that lactate is not inherently immunosuppressive or activating but rather functions as a metabolic modulator whose effect is tightly governed by cellular context, differentiation status, and metabolic readiness. A single molecule can either sustain effector activity or reinforce stem-like memory programs depending on the cell’s redox environment, transporter usage, and epigenetic accessibility.

These observations underscore that lactate’s immunoregulatory effects are inherently context-dependent, shaped by cellular metabolic programs, transporter dynamics, and redox balance. As such, this complexity imposes fundamental challenges for therapeutic targeting of lactate metabolism: interventions aimed at broadly reducing lactate levels or blocking lactate transport could produce divergent, even opposing, immunologic outcomes depending on immune cell type, metabolic state, and microenvironmental context [109].

Several lactate-targeting agents have nevertheless advanced into clinical or preclinical evaluation. AZD3965, a selective MCT1 inhibitor, has completed a phase I trial (NCT01791595) in patients with advanced solid tumors and lymphomas, demonstrating pharmacodynamic target engagement and acceptable safety in tumors characterized by high MCT1 and low MCT4 expression [110]. Preclinical studies further show that AZD3965 inhibits tumor growth and increases infiltration of dendritic cells and NK cells. Tumor-derived lactate has been shown to impede the binding of PD-L1 antibodies, contributing to resistance against PD-1/PD-L1 blockade [111]. Notably, combined treatment with AZD3965 and a PD-L1 antibody–drug conjugate (PD-L1-ADC) effectively overcame this resistance in preclinical models, improving antitumor responses [111].

In parallel, LDHA inhibitors such as FX11 and galloflavin have demonstrated efficacy in preclinical models of lymphoma, pancreatic, and breast cancers by reducing lactate production, disrupting glycolysis, and inducing oxidative stress [112,113]. While direct GPR81 antagonists remain in preclinical development, their ability to reverse lactate-driven immunosuppression in breast and other tumor models reinforces the therapeutic value of targeting lactate signaling pathways [49].

Together, these findings highlight the promise of integrating lactate metabolism inhibitors into cancer immunotherapy. However, therapeutic success will require precision approaches that account for differences in metabolic programming among tumor cells and immune subsets, careful selection of molecular targets (e.g., LDHA, MCT1, GPR81), and strategies to minimize potential adverse effects on nonmalignant tissues. These considerations underscore the translational challenge of exploiting lactate biology for therapeutic gain while maintaining immune and metabolic homeostasis.

As a potential strategy to improve the accuracy and efficacy of these therapeutic approaches, recent advances in spatial metabolomics have significantly enhanced our understanding of how lactate gradients contribute to the heterogeneity of the tumor microenvironment [114]. Techniques such as MALDI-MSI and DESI-MSI enable high-resolution mapping of metabolite distributions within tumor tissues, revealing how localized metabolic environments shape immune cell behavior [115]. Spatially resolved immunofluorescence and image analysis have demonstrated that hypoxia and lactate gradients within tumors generate distinct tumor-associated macrophage (TAM) phenotypes: ARG1^+^ macrophages preferentially accumulate in hypoxic, lactate-rich regions, whereas MRC1^+^ macrophages are enriched near blood vessels in normoxic areas (r = 0.6 and −0.2 correlation coefficients, respectively; *p* < 10) [116]. This example underscores the importance of integrating spatial metabolomics to resolve tumor- and immune context-specific heterogeneity and to guide precision therapeutic strategies targeting lactate metabolism.

Collectively, these insights position lactate as a central integrator of immune cell fate. Its capacity to influence redox balance, substrate competition, transcriptional reprogramming, and chromatin modification underscores the necessity for therapeutic approaches that account for cell-type specificity and the complexity of the microenvironment. Rather than viewing lactate as a singular target, future strategies must consider it as a dynamic rheostat within immunometabolic networks: one that is capable of tipping immune responses toward activation or suppression depending on nuanced intracellular and extracellular cues.

## 4. Conclusions

Lactate functions as a dynamic immunometabolic modulator whose effects depend on its source, concentration, and cellular context. Rather than acting through a single mechanism, lactate integrates transport, signaling, metabolic, and epigenetic pathways to influence immune cell fate and function. Understanding this complexity will be essential for developing therapeutic strategies that target lactate-related pathways without disrupting immune homeostasis. Future interventions must account for cell-type-specific metabolic programming, as well as the dual roles of lactate as both a nutrient and a signaling molecule, to achieve selective immune modulation.

## Figures and Tables

**Table 1:** Expression of monocarboxylate transporters and their roles in immune cells.

Cell Type	MCT1	MCT4	Metabolic Characteristics	Immunologic Consequences	References
Effector T cells	↑	↑	Glycolysis ↑ lactate export ↑	Supports rapid proliferation and effector cytokine production	[27,28]
Regulatory T cells	↑	↓	Lactate uptake ↑ Oxidative metabolism ↑	Enhances survival and suppressive function in acidic environments	[29,30]
Activated B cells	↑	-	Glycolysis ↑ lactate export ↑	Supports proliferation and antibody production	[31]
M1 Macrophages	↓	↑	Glycolysis↑ Lactate export ↑	Promotes pro-inflammatory phenotype and cytokine secretion	[32]
M2 Macrophages	↑	↓	Lactate uptake ↑ Oxidative metabolism ↑	Facilitates expression of anti-inflammatory genes	[32]
Dendritic cells	↑	-	Lactate uptake ↑ Oxidative metabolism ↑	Facilitates expression of anti-inflammatory genes	[33]
